# Disruptions in the Cystic Fibrosis Community’s Experiences and Concerns During the COVID-19 Pandemic: Topic Modeling and Time Series Analysis of Reddit Comments

**DOI:** 10.2196/45249

**Published:** 2023-04-20

**Authors:** Lean Franzl Yao, Kiki Ferawati, Kongmeng Liew, Shoko Wakamiya, Eiji Aramaki

**Affiliations:** 1 Social Computing Laboratory Nara Institute of Science and Technology Ikoma Japan

**Keywords:** COVID-19, Reddit, time series analysis, BERTopic, topic modeling, cystic fibrosis

## Abstract

**Background:**

The COVID-19 pandemic disrupted the needs and concerns of the cystic fibrosis community. Patients with cystic fibrosis were particularly vulnerable during the pandemic due to overlapping symptoms in addition to the challenges patients with rare diseases face, such as the need for constant medical aid and limited information regarding their disease or treatments. Even before the pandemic, patients vocalized these concerns on social media platforms like Reddit and formed communities and networks to share insight and information. This data can be used as a quick and efficient source of information about the experiences and concerns of patients with cystic fibrosis in contrast to traditional survey- or clinical-based methods.

**Objective:**

This study applies topic modeling and time series analysis to identify the disruption caused by the COVID-19 pandemic and its impact on the cystic fibrosis community’s experiences and concerns. This study illustrates the utility of social media data in gaining insight into the experiences and concerns of patients with rare diseases.

**Methods:**

We collected comments from the subreddit r/CysticFibrosis to represent the experiences and concerns of the cystic fibrosis community. The comments were preprocessed before being used to train the BERTopic model to assign each comment to a topic. The number of comments and active users for each data set was aggregated monthly per topic and then fitted with an autoregressive integrated moving average (ARIMA) model to study the trends in activity. To verify the disruption in trends during the COVID-19 pandemic, we assigned a dummy variable in the model where a value of “1” was assigned to months in 2020 and “0” otherwise and tested for its statistical significance.

**Results:**

A total of 120,738 comments from 5827 users were collected from March 24, 2011, until August 31, 2022. We found 22 topics representing the cystic fibrosis community’s experiences and concerns. Our time series analysis showed that for 9 topics, the COVID-19 pandemic was a statistically significant event that disrupted the trends in user activity. Of the 9 topics, only 1 showed significantly increased activity during this period, while the other 8 showed decreased activity. This mixture of increased and decreased activity for these topics indicates a shift in attention or focus on discussion topics during this period.

**Conclusions:**

There was a disruption in the experiences and concerns the cystic fibrosis community faced during the COVID-19 pandemic. By studying social media data, we were able to quickly and efficiently study the impact on the lived experiences and daily struggles of patients with cystic fibrosis. This study shows how social media data can be used as an alternative source of information to gain insight into the needs of patients with rare diseases and how external factors disrupt them.

## Introduction

The COVID-19 pandemic was the first global pandemic since the invention of Facebook [1,2]. As lockdowns and quarantine protocols were put in place, the use of social media to spread information rose, providing an abundance of social media data and even leading to an infodemic [3,4]. Needless to say, people’s day-to-day lives drastically changed during this period in dealing with the threat and risks posed by COVID-19, and all of these were recorded in social media [5,6].

Compared to the general population, patients with cystic fibrosis were also faced with the risk of overlapping symptoms with COVID-19 in addition to the challenges already faced by patients with rare diseases that preceded the global pandemic [7,8]. Cystic fibrosis is a condition caused by a mutation in a gene that affects a person’s production of mucus and sweat and commonly causes pulmonary or respiratory problems; depending on the types of mutations, there can be different symptoms and treatment options [9,10]. It is possible that some measures already taken by the cystic fibrosis community help mitigate the risk imposed by COVID-19, such as ongoing treatments or the added caution that comes with living with cystic fibrosis [11]. However, health measures and protocols raised to deal with the COVID-19 pandemic also disrupted clinic visits, medication, and therapy, among others, in addition to the challenges faced by the general population.

For the cystic fibrosis community and also other rare disease communities, social media has been a source of information and support even before the global pandemic [12,13]. Researchers have even used this available information to gain insight into patients and communities with rare diseases [14,15]. Combined with natural language processing techniques, natural dialogue on social media platforms can provide useful insight into patients’ daily lives. Reddit, for example, is one platform used by patients with rare diseases, which is also used in medical research. Foufi et al [16] analyzed chronic diseases using extracted entities and relations from Reddit discussions. Leung et al [17] explored COVID-19–related stressors by applying topic modeling to Reddit data. In Zhu et al’s [18] study, they illustrated how we could gain insight into the needs of patients with rare diseases by analyzing social media data. They found popular rare disease subreddits and did a case study on the subreddit r/CysticFibrosis. Topic modeling has also been used in other data sets to gain insight into patients’ needs [19,20].

Studying social media data of communities with rare diseases provides a ground-up level point of view from patients’ personal experiences and concerns that may not otherwise be shared with medical care providers. Using social media data also has the added benefit of speed since collecting data does not require additional time from patients. Because the use of social media platforms by these communities predates the pandemic, the available data can provide information about the disruptions caused by the pandemic in these communities. In this study, we propose to look at the disruption caused by COVID-19 on the discussion topics of patients with cystic fibrosis through the use of time series analysis and topic models.

## Methods

### Data Collection and Preprocessing

We collected comments from the subreddit r/CysticFibrosis to represent the experiences and concerns of the cystic fibrosis community. On August 31, 2022, we collected all Reddit comments starting from the inception date of March 24, 2011, using the Pushshift Reddit application programming interface through Python (version 3.9.12; Python Software Foundation). The Pushshift Reddit application programming interface offers a convenient way to collect text data for all comments and submissions in a chosen subreddit. We made this data set, as well as the script we used to collect the data, available on our GitHub repository [21].

We checked for duplicate comments and converted everything to lowercase. We also removed links, tags, and mentions of other users. At this stage, we did not perform any further data cleaning to maintain the natural structure of the comments since the BERTopic library was developed with natural text and has its own way of dealing with noise and outliers.

### Topic Modeling

BERTopic is a topic modeling technique that uses state-of-the-art language models and applies a class-based term frequency-inverse document, which calculates how relevant a word is to the class of documents and uses a frequency procedure for generating topics [22]. We trained a BERTopic model on the Reddit comments using the library bertopic (version 0.12.0 [22]). To keep our results consistent and reproducible with each iteration, we set the random state parameter of the Uniform Manifold Approximation and Projection model to 42 using the library umap (version 0.0.1 [23]). With these parameter specifications, BERTopic produced 824 topic categories: 823 usable topics and a topic for outliers, topic “–1.” Additional cleaning was done to remove stopwords by defining a vectorizer with sklearn (version 3.7 [24]) and setting the stopwords using the list from spacy (version 3.3.1 [25]). We also included “im,” “like,” “use,” “ive,” and “ill” to the list of stopwords before finally reducing the number of topics further.

We set BERTopic to reduce the number of topics from 823 to 30 and noticed that most of the comments from the dropped topics were classified as outliers rather than belonging to one of the 30 topics. Instructing BERTopic to produce 30 topics from the beginning similarly resulted in more outliers. Thus, in order to mitigate the loss of data and obtain more general topics, we set BERTopic to reduce the number of topics to 30 and then used hierarchical clustering to manually merge topics based on topic similarity metrics.

### Time Series Analysis

#### Overview

To check the significance of an event on time series data, we used tools available in time series analysis. We used R (version 4.2.0; R Core Team) and the packages *tidyverse*, *tseries*, *TSA*, and *lmtest* for the time series analysis.

To determine whether COVID-19 was a significant predictor of the number of monthly comments, we prepared data sets that contain the number of monthly comments and the number of monthly active users. We prepared data sets to look at the overall activity and also at the individual topics. We took a denoised aggregate subset, that is, disregarding topic –1, to represent the overall activity. Likewise, for each topic, we took the subset of comments that belong to it. We then fit an autoregressive integrated moving average (ARIMA) model on each of these data sets: the denoised aggregate data set and the data sets for each topic. In addition, we also decided to check the significance of COVID-19 on the number of active users per month.

#### ARIMA(p,d,q) Model With Exogenous Variables

We fitted an ARIMA(*p*,*d*,*q*) model for each data set [26]. We created a dummy variable to indicate the period of the COVID-19 pandemic. A value of 1 was assigned for the months of January to December 2020, while the months of March 2011 to December 2019 and January 2021 to August 2022 were assigned a value of 0. We chose this start date of the pandemic period based on the month when the first case of COVID-19 was recorded and decided the end date to be the end of 2020 as vaccines and relaxations to health protocols were starting [27]. To account for the influence of the number of active users, we incorporated it as an exogenous variable in the model. This was to ensure that we were isolating the effects of each variable, particularly that of the period of COVID-19.

An ARIMA(*p*,*d*,*q*) model takes 3 parameters: the order of the autoregressive (AR) process (*p*), the number of times to difference the data in order to make it stationary (*d*), and the order of the moving average (MA) process (*q*). Stationarity is an assumption regarding the structure of a stochastic process like time series data; a stationary process is similar to saying that the behavior of the system governing the process does not change. In many cases, taking the differenced data and taking the subtracted value at successive timesteps, is enough to satisfy this assumption. An AR process describes a series wherein the value at a timestep can be described by its previous values (lagged values), while an MA process describes a series wherein the value at a timestep can be described by the lagged residual errors. Fitting an ARIMA model requires checking the stationarity of the data, determining whether it follows an AR, MA, or mixed ARMA process, and then deciding on the order.

We use the graph of the auto-correlation function (ACF) and the Augmented Dickey-Fuller unit root test to check for stationarity and determine the number of differences needed to make the data stationary. We then graph the ACF and partial auto-correlation function (PACF) together to see whether we would proceed to use an AR, MA, or a mixed ARMA. Although it was not needed in this study, we mention here that the order for an ARMA process is decided based on the extended auto-correlation function. Table 1 shows how the orders are determined based on the observed behaviors in the ACF and PACF. We also took a conservative stand in deciding the order, opting for lower orders unless the graph compellingly indicated a higher order.

**Table 1:** Behavior of auto-correlation function (ACF) and partial auto-correlation function (PACF) for autoregressive (AR), moving average (MA), and ARMA processes.

	AR (order *P*)	MA (order *q*)	ARMA
ACF	Tails off (decays to zero)	Cuts off after lag *q*	Tails off
PACF	Cuts off after lag *P*	Tails off (decays to zero)	Tails off

In time series models, incorporating another time series as an exogenous variable requires additional steps. We first look at the cross-correlation of the prewhitened time series of the number of active users and the number of comments to see which lags are correlated, and then decide on the transfer function to be used to add the exogenous variable to the ARIMA model. We prewhiten the time series data in order to remove linear trends and autocorrelation within each time series to avoid misleading cross-correlations. The cross-correlations then tell us which lags in the time series for the number of users are correlated with the time series for the number of comments.

#### Significance Testing

Once a model has been chosen and fitted to the time series data, we test for the significance of the dummy variable included in the model. The test for significance tells us whether there was a statistically significant change in trend during the COVID-19 period, and the sign of the coefficient tells us whether the effect was an increase or decrease in the number of comments.

### Ethical Considerations

This study did not require participants to be involved in any physical or mental intervention. As this research did not use personally identifiable information, it was exempt from institutional review board approval in accordance with the Ethical Guidelines for Medical and Health Research Involving Human Subjects stipulated by the Japanese national government.

## Results

### Collected Data

**Figure 1:**
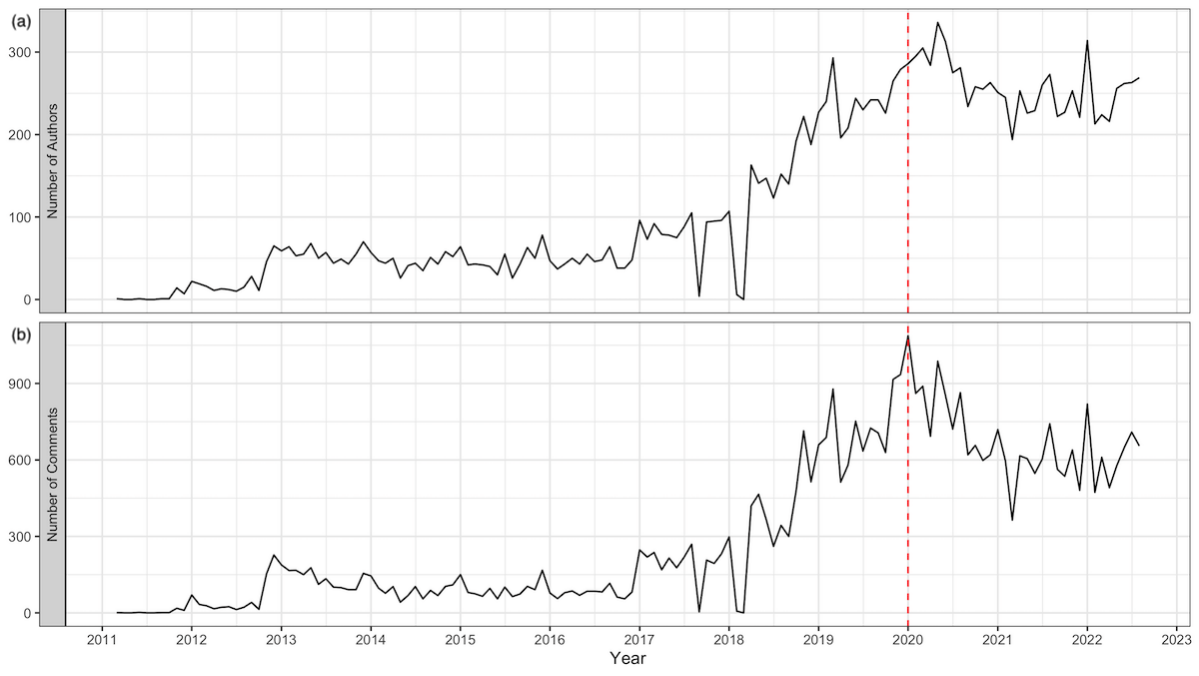
Timeline of monthly active users (A) and monthly comments (B) from March 24, 2011, to August 31, 2022. A red dotted line indicates the date when the World Health Organization declared COVID-19 a public health emergency of international concern. Both timelines show similar trends over the time period. There was a notable surge in active users and the number of comments at the start of 2018, but then it slowed down and started to decrease at the beginning of COVID-19.

A total of 120,738 comments from 5827 unique user IDs were collected, with dates ranging from inception on March 24, 2011, until August 31, 2022. Figure 1 shows the number of comments and the number of users per month. Table 2 contains sample comments and their assigned topics.

**Table 2:** Sample comments and their assigned topics.

Comment	Topic
Better than in USA. Here we need to renew Trikafta pre-authorization every 6 months. And if you lose your job and private insurance coverage, you’re screwed!	Trikafta and side effects
Those results sound amazing!! I’m so excited to try this now! Thank you!!	Gratitude
You’re going to get a whole spectrum of people with different levels of acceptable risk. Some people refuse to ever be in the same room as another CF’er while others sleep together. But yes, I’d agree his handling of this is concerning. Like, what does the world need to look like for him to decide its safe enough to go out again?	Social life
Mine had to be done on my upper arm where there was no hair to interfere with the collection. My son’s failed to collect any sweat, it’s very common.	Sweat testing and mutations
You can get it at a pot shop. You can use it as an edible too. I was told if you put a couple of pumps in a drink with a lot of fat (hot chocolate made with milk, milkshakes) it works a lot faster. I take it before bed. It helps with my anxiety and the muscle pain in my neck and back.	Marijuana
I dont think I have I just a nose spray that makes me be able to breathe easier with my nose	Sinuses and breathing

### Identified Topics

**Figure 2:**
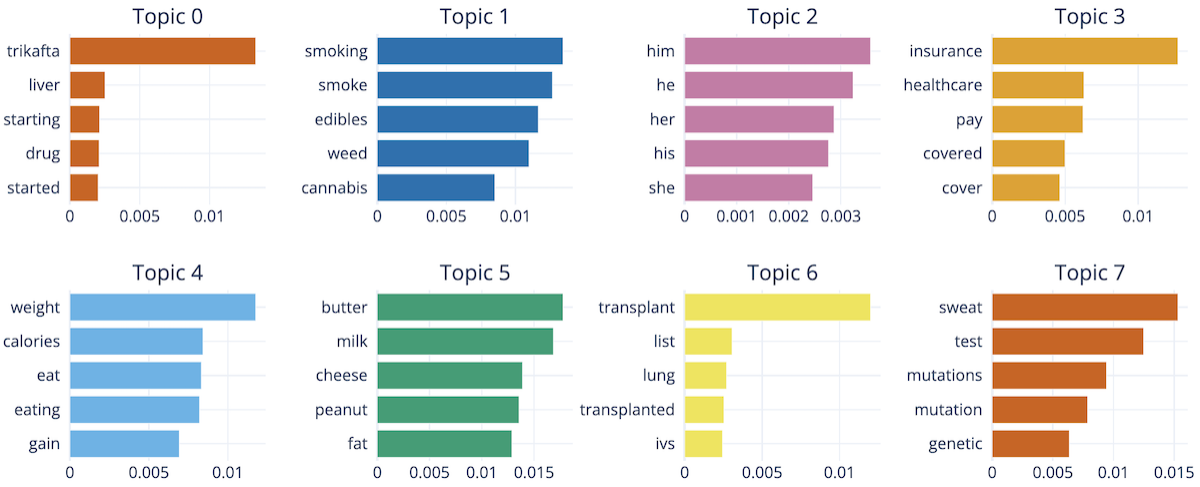
The top 5 representation words based on class-based term frequency-inverse document (c-TF-IDF) scores for the top 8 topics. The c-TF-IDF score represents how relevant a word is in representing the topic, with a higher score meaning that the word is more relevant. In topic 0, the word “trikafta” has a high score compared to the other top words, meaning that the topic representation relies on the presence of the word “trikafta” the most, while other topics have a more equal weighted representation, like topic 7.

**Figure 3:**
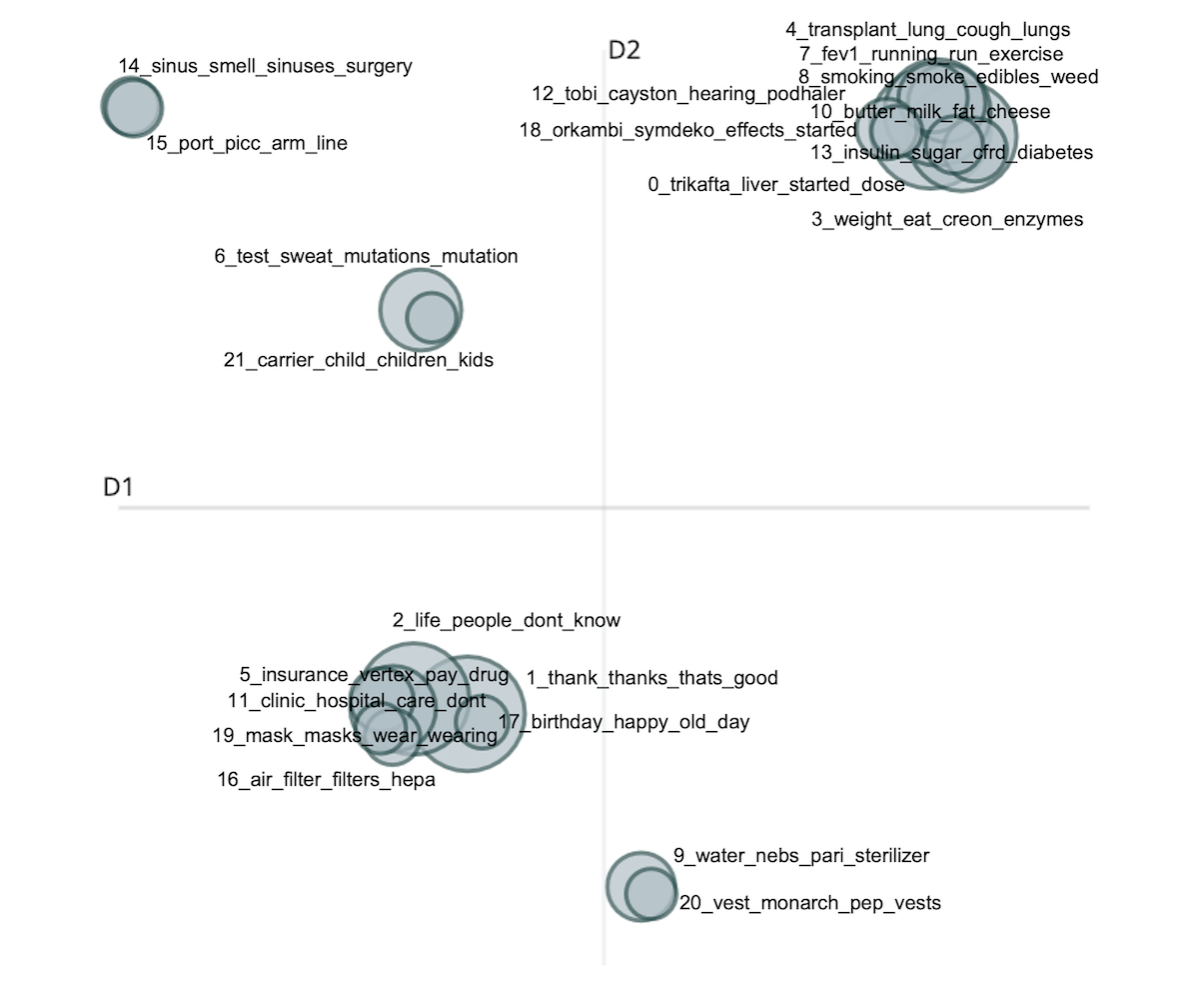
A 2D graph of the intertopical distance map using the embedded class-based term frequency-inverse document (c-TF-IDF) representations of the topic. The size of the circles represents how many documents were classified into that topic, and the x- and y-axes have no meaning or interpretation, but the distance between topics represents the similarity between them, where more similar topics are closer to each other. This plot can give an idea of how topics can be clustered together to form more general topics.

The initial 823 topics were too much for us to analyze. We specified our BERTopic model to reduce the number of topics to 30 instead, as a more manageable starting point. Figure 2 shows the top 8 topics and the words with the highest class-based term frequency-inverse document scores representative of each topic, and Figure 3 shows the intertopical distance map between topics that can be used to create more general topics.

**Figure 4:**
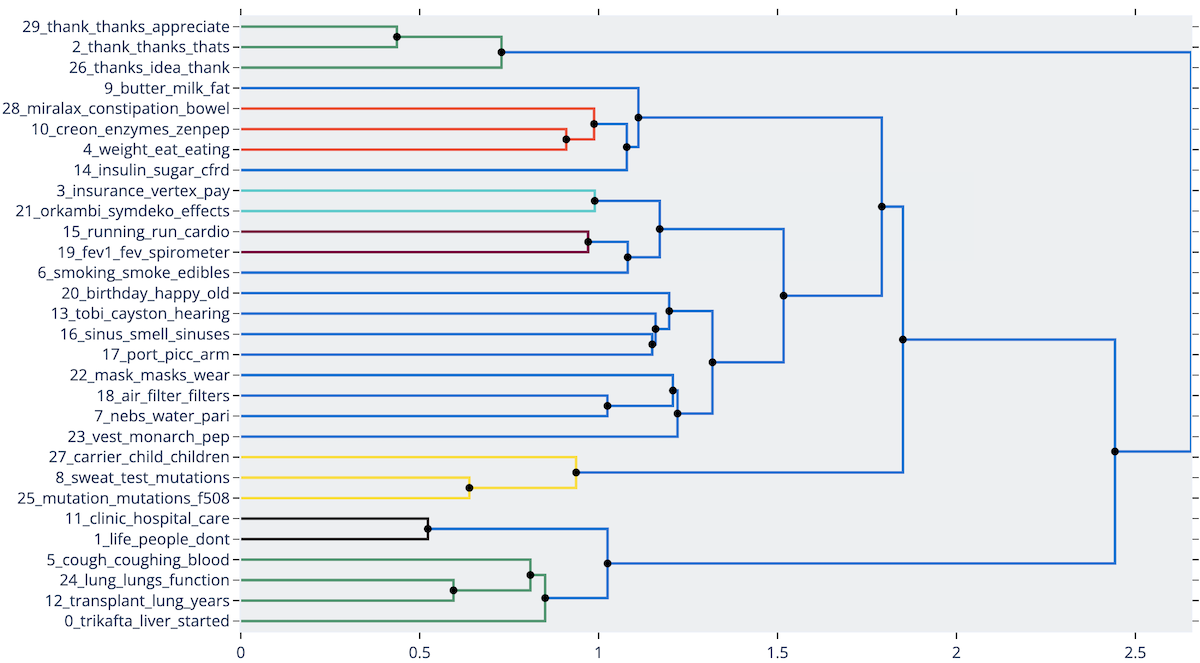
BERTopic hierarchical clustering. The hierarchical clustering shows how topic embeddings can be merged at different varying cosine distances. The figure also provides recommended clusters to merge and shows the topics in the same color, where blue is the default color and is not treated as a cluster.

A dendrogram graph was generated to visualize the hierarchical clustering of the 30 topics to see which ones were similar enough to be merged (Figure 4). The clustering was based on the cosine distance matrix of the topic embeddings with default parameters in BERTopic. Changing these parameters will result in different, more, or fewer cluster suggestions, so we merely use this result as a guide to manual merging.

In each cluster, we merged similar topics based on our judgment and understanding of what these topics discussed. We treated the dendrogram from BERTopic as recommendations on which topics we could look into for merging. From these recommended clusters, we looked at representative comments and also the top 10 representative words for the topic to make our decisions regarding merging. In addition, we looked into symptoms, treatments, life expectancy, comorbidities, and the general lives of patients with cystic fibrosis in order to name each of the merged topics. We decided to merge the following sets of topics: {29, 2, 26}, {28, 10, 4}, {15, 19}, {8, 25}, and {5, 24, 12}. In contrast, we chose not to merge the set {3, 21} since one appears to talk about financing while the other was about some type of medication; similarly, we excluded 27 from {8, 25} since the keywords for topic 27 imply that it talks about children and hereditariness, while 8 and 25 talks about mutations; likewise, topics 11 and 1 both have keywords that talk about medical care and life in general; and lastly, topic 0 was not included in {5, 24, 12} because it looks like topic 0 talks about a specific drug, Trikafta, and the liver, while the others talked about things related to respiration or the respiratory tract. After this step, we were left with 42,060 comments categorized into 22 topics. In Table 3, we summarize these topics, their top 10 representative words, and how they were merged relative to the original 30 topics.

**Table 3:** Top 10 representative words for merged topics based on the class-based term frequency-inverse document scores.

Merged topics	Top 10 representative words	Original topics
Trikafta and side effects	trikafta, liver, started, dose, dont, taking, effects, drug, feel, day	0
Gratitude	thank, thanks, thats, good, awesome, great, luck, oh, hear, congrats	2, 26, 29
Social life	life, people, dont, know, time, think, things, feel, want, going	1
Creon medication and diet	weight, eat, creon, enzymes, eating, food, fat, calories, diet, gain	4, 10, 28
Lung transplants and respiration	transplant, lung, cough, lungs, coughing, blood, function, time, mucus, dont	5, 12, 14
Vertex treatment and financing	insurance, vertex, pay, drug, copay, price, cost, drugs, health care, company	3
Sweat testing and mutations	test, sweat, mutations, mutation, genetic, symptoms, rare, testing, gene, diagnosed	8, 25
FEV_1_^a^ marker for CF^b^	fev1, running, run, exercise, cardio, good, bike, time, gym, dont	15, 19
Marijuana	smoking, smoke, edibles, weed, cannabis, thc, marijuana, vaping, cbd, smoked	6
Nebulizers	water, nebs, pari, sterilizer, eflow, bottle, neb, nebulizer, compressor, baby	7
Dairy and calories	butter, milk, fat, cheese, peanut, cream, eat, calories, protein, food	9
Medical facilities and professionals	clinic, hospital, care, dont, doctor, know, nurses, nurse, time, team	11
Tobramycin side effects and alternatives	tobi, cayston, hearing, podhaler, loss, voice, colistin, tobramycin, month, inhaled	13
Diabetes	insulin, sugar, cfrd, diabetes, blood, sugars, glucose, pump, diabetic, diet	14
Sinuses and breathing	sinus, smell, sinuses, surgery, nose, rinses, nasal, ent, polyps, surgeries	16
IV^c^ access port	port, picc, arm, line, veins, lines, piccs, ivs, dressing, iv	17
Air quality	air, filter, filters, hepa, live, purifier, house, humidity, ac, purifiers	18
Birthdays	birthday, happy, old, day, ich, year, und, mit, years, months	20
Orkambi medication and alternatives	orkambi, symdeko, effects, started, better, kalydeco, didnt, taking, function, year	21
Masks	mask, masks, wear, wearing, n95, surgical, people, cambridge, protect, vogmask	22
Airway clearance vests	vest, monarch, pep, vests, effective, clearance, flutter, aerobika, acapella, hillrom	23
CF inheritance and family planning	carrier, child, children, kids, chance, pregnant, carriers, pregnancy, birth, baby	27

^a^FEV_1_: forced expiratory volume in 1s.

^b^CF: cystic fibrosis.

^c^IV: intravenous.

**Figure 5:**
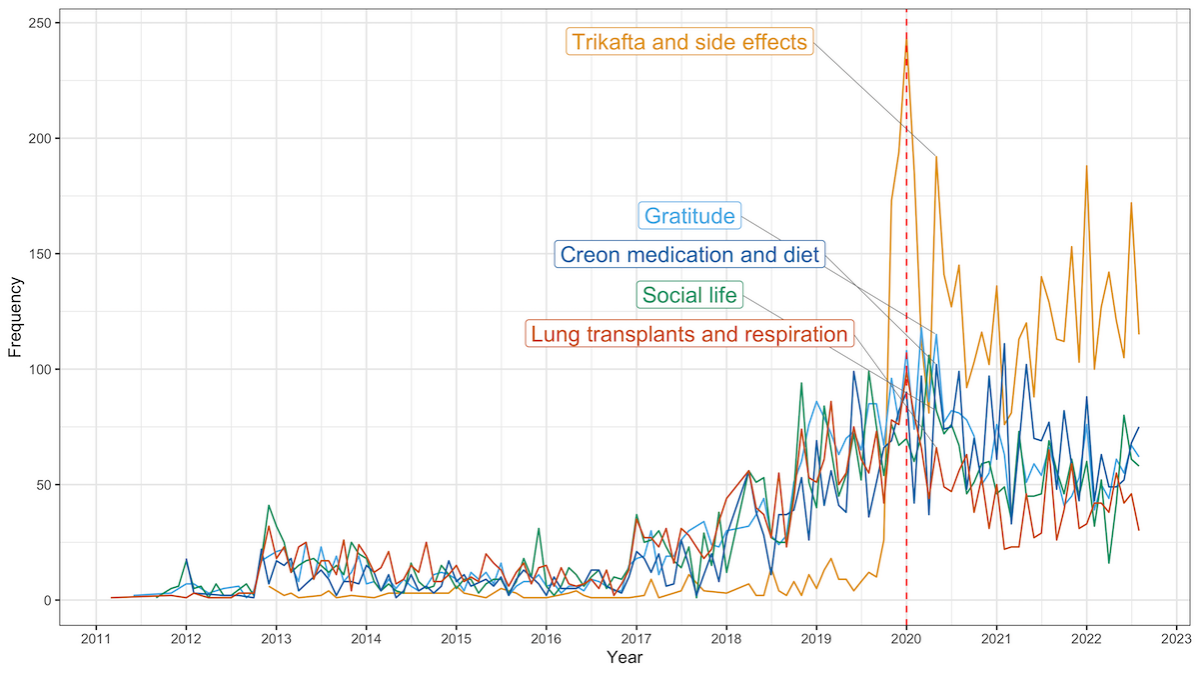
Timeline for the monthly comments per topic for the top 5 topics. A red dotted line indicates the date when the World Health Organization declared COVID-19 a public health emergency of international concern. There was also a surge in the number of comments at the start of 2018, but it slowed down and started to decrease at the beginning of the COVID-19 pandemic in 2020.

While it was not our main focus of the analysis, we provided a simple visualization of the evolution of topics within our time period using dynamic topic modeling. Figure 5 shows the time series of the top 5 topics. From the graph, we can see that there was little activity in the early years of the subreddit, and most topics seemed to be fairly uniform until around 2020 when more variations between topics became visible.

### Time Series Analysis

We first checked the cross-correlation of the prewhitened series for the number of active users and the number of comments and noticed that only lag 0 was significant. This means that both time series are correlated within the same time period rather than timesteps ahead of or behind each other. To simplify the inclusion of the number of active users as a regressor, we add it to the regression model in the same way a regressor would be added to a linear regression model without the use of a transfer function.

The series for each data set were nonstationary after observing the graphs of their ACFs. We determined that 1 order of differencing was enough to make each series stationary and confirmed this with the Augmented Dickey-Fuller unit root test. Observing the behaviors of their PACFs, which cuts off after a certain lag in addition to their ACF decaying to zero (see Table 1), leads us to conclude that each of the series follows an AR process. We determine the orders for the AR process by the lag where the PACF cuts off (see Table 4). The order of the AR process can be interpreted as how many lags are predictive of the current timestep; for example, an AR(3) process implies that the observation at the current timestep can be described using observations from the past 3 timesteps. We also note here that for the series representing the topic “IV access port,” the cutting-off behavior of the PACF was not as apparent as in the other series, but we still opted to fit an AR process for simplicity.

**Table 4:** Data sets, time series model specifications, and significance of the COVID-19 period.

Data set	Comments	ARIMA^a^ order	Coefficient of COVID-19 variable	*P* value
Aggregate data	42,060	(1,1,0)	–157.185	<.001
Trikafta and side effects	4705	(1,1,0)	–18.765	<.001
Gratitude	4240	(1,1,0)	–9.980	.004
Social life	4008	(1,1,0)	4.093	.10
Creon medication and diet	3876	(2,1,0)	3.371	.34
Lung transplants and respiration	3654	(1,1,0)	–6.566	.01
Vertex treatment and financing	2370	(1,1,0)	–5.087	.11
Sweat testing and mutations	2118	(1,1,0)	–5.468	.006
FEV_1_^b^ marker for CF^c^	1952	(1,1,0)	0.332	.91
Marijuana	1551	(1,1,0)	4.808	.039
Nebulizers	1487	(2,1,0)	–0.937	.61
Dairy and calories	1302	(4,1,0)	0.365	.58
Medical facilities and professionals	1299	(2,1,0)	–2.543	.003
Tobramycin side effects and alternatives	1187	(3,1,0)	–3.641	.003
Diabetes	1061	(4,1,0)	–0.493	.67
Sinuses and breathing	1037	(2,1,0)	0.563	.53
IV^d^ access port	1036	(1,1,0)	1.809	.30
Air quality	1001	(2,1,0)	–1.243	.15
Birthdays	899	(2,1,0)	–1.264	.09
Orkambi medication and alternatives	840	(1,1,0)	–1.247	.39
Masks	828	(1,1,0)	0.368	.78
Airway clearance vests	819	(2,1,0)	1.239	.12
CF inheritance and family planning	790	(1,1,0)	–3.029	.002

^a^ARIMA: autoregressive integrated moving average.

^b^FEV_1_: forced expiratory volume in 1s.

^c^CF: cystic fibrosis.

^d^IV: intravenous.

The results from fitting an ARIMA model for the number of comments with exogenous variables for the number of active users and the dummy variable to indicate the months of the COVID-19 pandemic can be seen in Table 4. For all series, all lags following the orders for the AR process were significant at an α level of .05.

The last column in Table 4 shows the *P* values for the COVID-19 dummy variable. Using an α level of .05, we say that the user activity for the data sets that show a *P* value less than .05 showed a significant difference in trend during the COVID-19 pandemic. There were 9 data sets: the denoised aggregate data set and 8 topic data sets that showed a significant change in trend. For the aggregate data set and topics “Trikafta and side effects,” “Gratitude,” “Lung transplants and respiration,” “Sweat testing and mutations,” “Medical facilities and professionals,” “Tobramycin side effects and alternatives,” and “CF inheritance and family planning,” there was a significant decrease in the number of comments during the COVID-19 period. In contrast, for the topic “Marijuana,” there was a significant increase in the number of comments during the period.

We followed the same steps to check the significance of COVID-19 on the number of active users without any exogenous variables except for the dummy variable representing COVID-19 and found that COVID-19 was not statistically significant in the model. This implies that the trend in activity during the pandemic period was not significantly different from the trend outside this period.

## Discussion

### Principal Results

There is a mix of increased and decreased activity for topics that showed a significant difference in trend during the COVID-19 pandemic. This indicates a shift in attention or focus of discussion topics during the pandemic, despite an overall decrease in activity on the subreddit. In addition, our results also describe the time series processes that describe user activity for each topic. These models show how immediate or prolonged the predictive capacity of past activity is for future activity.

The topics from our BERTopic model showed a variety of discussion interests within the cystic fibrosis community, ranging from medications, treatments, symptoms, finances, or living with their disease. By incorporating a dummy variable for the pandemic period, we were able to check the statistical significance of this period on the amount of user activity for each topic.

Our study shows that there was an overall decrease in user activity during the pandemic. Considering that there was no statistically significant decrease in the number of active users during the pandemic, the decrease in user activity during this period can be attributed to other factors, such as other priorities that came about during the pandemic. Users could have been spending less time commenting on subreddits and instead focusing on gathering information about the pandemic, keeping up with changing health protocols, job security, or family members, to name a few. The decreased activity during this period did not automatically reflect in the individual topics, as evident in the increased activity for the topic “Marijuana.” In the succeeding subsections, we discuss the topics that underwent a significant change in trend during the pandemic and the possible reasons behind the change, as well as the varying ARIMA orders among the topics.

### Topics Significantly Affected by COVID-19

Column “*P* value” in Table 4 summarizes the significance testing for the COVID-19 dummy variable using an α level of .05. We interpret topics with a *P* value less than .05 for the dummy variable as showing a significant change in trend during the COVID-19 pandemic. In this section, we discuss the topics that showed a statistically significant difference in trends during the COVID-19 pandemic.

The topic “Marijuana” showed top keywords such as “smoking,” “smoke,” “edibles,” and “weed.” Looking at some sample comments on this topic led us to conclude that the discussion is about the use of cannabis and alternative means of using cannabis aside from smoking (see Table 2). The rise in activity during this period could be due to the additional stresses brought about by the pandemic and, thus, the curiosity in exploring the use of medical marijuana. In Stephen et al’s [28] study, they showed that side effects were rare and mild, should there be any at all, and that the use of medical marijuana was effective in relieving symptoms of stress and pain.

A trickle-down effect on the overall negative effect on the aggregate data set could explain topics with a negative coefficient for COVID-19. The topics “Trikafta and side effects” and “Tobramycin side effects and alternatives” both talk about medications and could have lower activity because of other newer and more pressing matters during the pandemic that require more of their attention. In contrast, these medications have a more established database of information. Trikafta is an approved medication for patients with cystic fibrosis with the most common type of mutation that targets the underlying cause, while Tobramycin is an antibiotic that targets *Pseudomonas aeruginosa* infection in patients with cystic fibrosis [29]. The decrease in activity for “Gratitude” could be explained by having fewer things for users to be grateful for during the period or due to fewer interactions overall. The topics “Lung transplants and respiration,” “Sweat testing and mutations,” and “Medical facilities and professionals” relate to hospital or clinic visitation; decreased activity could be explained by lower visitation rates due to limited hospital capacity or precautions in place for patients with cystic fibrosis to avoid getting COVID-19 [11]. Lastly, for the topic “CF inheritance and family planning,” the decreased activity could be due to higher uncertainties about the future in light of the long pandemic period and lower interest in discussing this topic.

### Topics With Higher ARIMA Orders

In this section, we discuss the interpretations for the ARIMA models used in each topic data set and give possible insights into their time series behavior. The ARIMA orders for the different data sets mostly follow an ARIMA(1,1,0) process, including the aggregate data set. This means that these series require 1 order of differencing to make the series stationary and can be described using an AR(1) process. An AR(1) process implies that the observation of the current time period can be described using observations from the previous time period. We can interpret this as users having shorter terms for engagement since only the previous timestep is descriptive of current activities. In contrast, topics that were modeled with a higher AR order imply a longer engagement of users since longer durations of user activity describe the current timestep.

The topics “Dairy and calories” and “Diabetes” have the highest AR order, AR(4). The higher order can be explained by a longer duration of interest in discussions or a user following up on previous discussions. Compared to an AR(1) process, an AR(4) process means that activities from the previous 4 timesteps are descriptive of the activities in the current timestep. Both topics describe dietary concerns, which could explain the prolonged engagement since it takes time to observe changes or side effects from diet changes. The topic “Diabetes” is talking specifically about cystic fibrosis–related diabetes, which is the most common complication experienced by patients with cystic fibrosis, with around 50% of patients with cystic fibrosis developing cystic fibrosis–related diabetes by the age of 30; the high comorbidity rate could be another reason for the prolonged engagement. Additionally, the topic “Creon medication and diet” is another topic relating to the diets of patients with cystic fibrosis and is modeled using AR(2) and is also longer than most other topics. These topics relate to the nutrition and diet of patients with cystic fibrosis, which need to be carefully monitored to avoid complications and improve symptoms [30-33].

The topic “Tobramycin side effects and alternatives” has a high AR order as well, AR(3). Tobramycin is an antibiotic used to treat *P aeruginosa* infections in patients with cystic fibrosis. This infection is highly prevalent among patients with cystic fibrosis, often results in chronic infections after the first infection, and is resistant to antibiotics [34,35]. This infection can drastically reduce the quality of life and life span. The seriousness of this infection and its resistance to antibiotics could explain the longer durations in discussing this topic.

Interestingly, topics that were modeled using the AR(2) had a number of topics (5 out of 7) related to respiration and common symptoms: “Nebulizers,” “Medical facilities and professionals,” “Sinuses and breathing,” “Air quality,” and “Airway clearance vests.” The higher AR order could be explained by longer discussions about symptoms, procedures used to alleviate symptoms, and the time it takes to notice effects or side effects. Interestingly, the topic “Birthdays” also follows an AR(2) process, where the long process could be due to prolonged celebrations of reaching another milestone [36].

### Limitations

Many of the comments were not classified into any topics, as evident by the 78,678 comments categorized as outliers in topic –1. This can be expected in social media, especially for short texts and quick exchanges between users; thus, these comments do not have enough contextual information for the model to classify them. Although some comments, on their own, may not talk about a specific topic, they may still contribute to the sentiment of the topic discussed in the parent comments or the main post.

We chose BERTopic for its convenience and because it assigns each document to exactly 1 topic; however, we did not spend much time fine-tuning the model and tweaking its performance. We checked a few comments for each topic and saw that they were properly clustered with similar documents. While BERTopic has shown more robust performance in topic modeling of social media posts than other topic modeling methods [37], topic modeling is still a fairly subjective process. The results we presented demonstrate the possibility of using topic modeling with time series analysis to gain insight into the needs of patients with rare diseases rather than to evaluate the performance of BERTopic as a topic model.

Our definition of the dummy variable used to indicate the period of the pandemic was based on how long we believe the pandemic affected the number of comments, but the effects of the pandemic could have started later or could have ended sooner. We chose the year 2020 to capture the early influence of the pandemic when cases were starting to be reported and until the end of the year, when countries were starting to relax health protocols. Our time series modeling was also not as extensive to perform the same procedure for each data set, referring to our assumption that only the current lag for each topic’s data set is significant following the behavior of the aggregate data set. The purpose of the analysis was to fit a model to the time series data and check for the statistical significance of an event rather than forecasting future values; thus, we no longer performed residual analysis to evaluate our models and instead observed the graph of the fitted values superimposed on the raw data. This allowed us to observe the broader trends across all the discussion topics rather than being restricted to only a small data set of topics.

### Conclusions

We focused on the cystic fibrosis community because of the active Reddit users in r/Cystic Fibrosis and because the symptoms of cystic fibrosis overlap with those of COVID-19. We found that during the events of the COVID-19 pandemic, there was an overall decrease in user activity. Our time series analysis accounting for the number of active users showed a mix of positive and negative effects from COVID-19, indicating a shift in focus in discussion topics during this period. Additionally, the varying orders of the ARIMA models among the topics indicate that the user activity for some topics has a longer predictive capacity than others. These topics do not necessarily reflect the needs or interests of patients with cystic fibrosis or patients with rare diseases more generally. Still, our results show what patients were concerned about in the dynamic interactions between patients on the subreddit, as well as how they were disrupted by the COVID-19 pandemic.

We anticipate that this methodology and these results can be further developed and studied to be used in providing better care for patients with rare diseases by gaining a better understanding of their needs and how they are affected by external factors. In contrast with the traditional survey- or clinical-based methods, analyzing social media is much quicker while still providing useful insights into patients with rare diseases. Future research may consider extending this methodology to other rare disease subreddits, or even comparisons across multiple subreddits, as a quick and efficient source of information for the lived experiences of patients and their daily struggles and concerns.

## Abbreviations

ACF: auto-correlation function
AR: autoregressive
ARIMA: autoregressive integrated moving average
MA: moving average
PACF: partial auto-correlation function

## Data Availability

The data used in this study are available in our GitHub repository [21].
